# Efficient CuO/Ag_2_WO_4_ photoelectrodes for photoelectrochemical water splitting using solar visible radiation

**DOI:** 10.1039/d3ra00867c

**Published:** 2023-04-11

**Authors:** E. Mustafa, E. A. Dawi, Z. H. Ibupoto, A. M. M. Ibrahim, A. Elsukova, X. Liu, A. Tahira, R. E. Adam, M. Willander, O. Nur

**Affiliations:** a Department of Sciences and Technology, Linköping University, Campus Norrköping SE-601 74 Norrköping Sweden elfatih.mohammed.mustafa@liu.se elfatihmustafa@gmail.com; b Nonlinear Dynamics Research Centre (NDRC), Ajman University P. O. Box 346 United Arab Emirates; c Institute of Chemistry, University of Sindh 76080 Jamshoro Pakistan; d Department of Pharmaceutical Chemistry, Jazan University P. O. Box 346 Kingdom of Saudi Arabia; e Department of Physics, Chemistry and Biology, Linköping University SE-58183 Linköping Sweden; f Institute of Chemistry, Shah Abdul Latif University Khairpur Mirs 66020 Sindh Pakistan

## Abstract

Water splitting energy production relies heavily on the development of high-performance photoelectrochemical cells (PECs). Among the most highly regarded semiconductor materials, cupric oxide (CuO) is an excellent photocathode material. Pristine CuO does not perform well as a photocathode due to its tendency to recombine electrons and holes rapidly. Photocathodes with high efficiency can be produced by developing CuO-based composite systems. The aim of our research is to develop an Ag_2_WO_4_/CuO composite by incorporating silver tungstate (Ag_2_WO_4_) nanoparticles onto hydrothermally grown CuO nanoleaves (NLs) by successive ionic layer adsorption and reaction (SILAR). To prepare CuO/Ag_2_WO_4_ composites, SILAR was used in conjunction with different Ag_2_WO_4_ nanoparticle deposition cycles. Physicochemical characterization reveals well-defined nanoleaves morphologies with tailored surface compositions. Composite CuO/Ag_2_WO_4_ crystal structures are governed by the monoclinic phase of CuO and the hexagonal phase of Ag_2_WO_4_. It has been demonstrated that the CuO/Ag_2_WO_4_ composite has outstanding performance in the PEC water splitting process when used with five cycles. In the CuO/Ag_2_WO_4_ photocathode, water splitting activity is observed at low overpotential and high photocurrent density, indicating that the reaction takes place at low energy barriers. Several factors contribute to PEC performance in composites. These factors include the high density of surface active sites, the high charge separation rate, the presence of favourable surface defects, and the synergy of CuO and Ag_2_WO_4_ photoreaction. By using SILAR, silver tungstate can be deposited onto semiconducting materials with strong visible absorption, enabling the development of energy-efficient photocathodes.

## Abstract 2

Using the hydrothermal method followed by the SILAR method, synthesis of CuO/Ag_2_WO_4_-5 photocathode that is effective for PEC water splitting when illuminated by solar light is provided.

## Graphical abstract

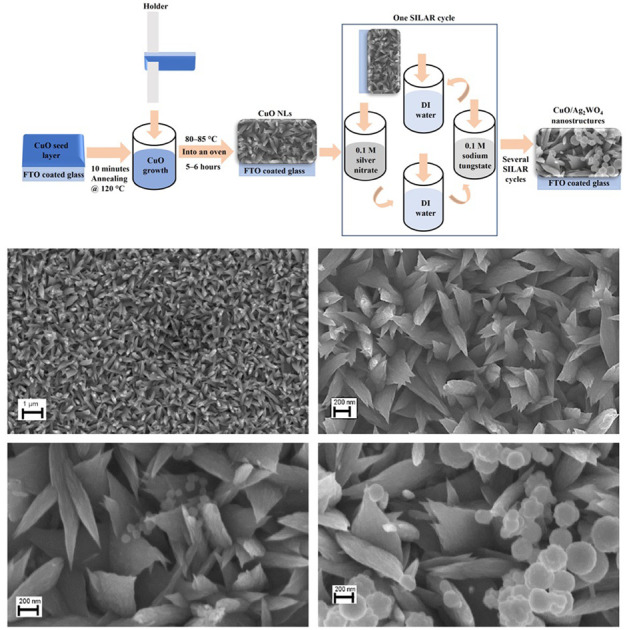

## 1 Introduction

The production of hydrogen from solar-powered water splitting has the potential to significantly strengthen renewable energy reservoirs. This is because it has a high density, clean energy carrier, abundant solar energy, and a low energy barrier.^1–4^ The phenomenon of water splitting is caused by two parallel reactions, namely the oxygen evolution reaction (OER) and the hydrogen evolution reaction (HER), which exhibit different kinetics in terms of activation energy. Particularly, the OER process is an energy-intensive reaction, which limits the overall efficiency of water splitting. This is the reason we have limited the portion of hydrogen production from water splitting to date.^5,6^ In view of the high energy requirement for water splitting, photoelectrochemical (PEC) water splitting is a versatile technology that is characterized by a low energy demand owing to its use of photons, and it allows the separation of two reactions easily, namely OER and HER.^7–11^

The choice of materials for fabricating photocathodes is based on their ability to absorb strong bands in the visible region, while also being low in cost, earth abundant, cost-effective, and environmentally friendly. There are a variety of semiconducting materials available, including n-type materials like TiO_2_, WO_3_, ZnO, Fe_2_O_3_, and BiVO_4_,^12–16^ as well as p-type materials like p-Si, Cu_2_O, CuO, p-InP, and p-CdS^17–23^ that have been examined for use as photoanodes and photocathodes for solar water electrolysis, respectively. Among these metal oxides, cupric oxide (CuO) is an exemplary photocathode material with many interesting features like narrow bandgap (1.3–1.7 eV), low cost, earth abundant, and easy to prepare by environment friendly methods.^19,24–28^ In addition to gas sensing, photocatalysis, lithium-ion batteries, and solar cells, CuO has been studied extensively for a wide range of applications.^29–32^ A further advantage of CuO is its high visible light absorption band, which makes it highly suitable for PEC water splitting.^33–36^ Due to the fast charge carrier recombination rate, CuO has relatively low photoactivity towards HER.^11,36,37^ To overcome the recombination rate, different techniques are utilized to amend the chemical composition and surface characteristics of CuO like reducing particle size and fabricating composite materials.^38,39^ There have been a variety of methods used to synthesize CuO nanostructures with various morphologies,^40^ among which CuO nanoleaves (NLs) grown by hydrothermal method is simple, inexpensive, scale up, and eco-friendly.^27,40^ In addition, silver-based semiconductors are considered suitable materials for increasing PEC water splitting efficiency.^41,42^ In recent years, the deposition of silver tungsten oxide or silver tungstate (Ag_2_WO_4_) nanoparticles by using a successive ionic layer adsorption and reaction (SILAR) method with various cycles onto metal oxide semiconductors with improved performance has been reported.^43–47^ The SILAR method allows controlling the thickness of the deposition based on solution concentration and cycle number up to micrometers.^48^ First, hydrothermal method was used to deposit nanoleaves of CuO. Low-cost, simple, scalable, eco-friendly, and environmentally friendly are some of the advantages of the hydrothermal method. The SILAR method was used due to the fact that it is a well-controlled method for producing uniform growth layers for the development of hybrid materials, as well as a cost effective method and requires little growth time. Based on these considerations, we used both of these methods in order to demonstrate photoelectrochemical water splitting using hybrid materials.

CuO/Ag_2_WO_4_ composites offer rapid charge transfer at the electrode–electrolyte interface by preventing electron and hole recombination charges. They also possess a tunable surface with a high density of active sites, resulting from various metallic oxidation states of Cu, Ag, and W, as well as unique oxygen surface defects, which enables advanced PEC water splitting. Composites of CuO with Ag_2_WO_4_ can also be used to demonstrate the effective transfer of charges at the interface. To our knowledge, no research has been conducted on the CuO/Ag_2_WO_4_ composite prepared by hydrothermal and SILAR methods in order to assess its performance in the PEC water splitting process.

Using hydrothermal and SILAR methods, we investigated the deposition of Ag_2_WO_4_ nanoparticles onto CuO NLs. Composites of CuO and Ag_2_WO_4_ have been studied using a variety of deposition cycles, including 5, 10, and 15, and its response to water splitting activity has been investigated. The CuO/Ag_2_WO_4_ composite has been examined by structural and photoelectrochemical characterization.

## 2 Experimental section

### 2.1. Synthesis of CuO/Ag_2_WO_4_ photoelectrodes

**Fig. 1:**
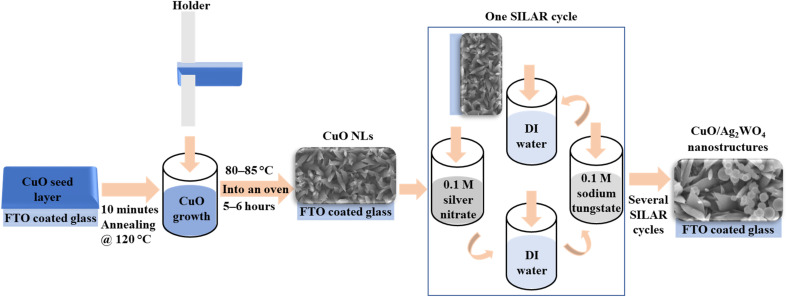
Schematic diagram showing the synthesis process of the CuO/Ag_2_WO_4_ composites. Preparation of CuO seed solution by drop-wise addition of 0.03 M potassium hydroxide (KOH) in 99% methanol to 0.01 M of copper(ii) acetate monohydrate Cu(C_2_H_3_O_2_)_2_·H_2_O in 99% methanol at 60 °C with stirring. For CuO growth, 0.05 M copper(ii) nitrate hemi(pentahydrate) (CuN_2_O_6_·2.5H_2_O) in 100 mL of deionized water was mixed with 0.025 M hexamethylenetetramine (HMT). The deposition of silver tungstate (Ag_2_WO_4_) nanoparticles (NPs) onto CuO nanoleaves (NLs) was achieved using the successive ionic layer adsorption and reaction (SILAR) method with different cycles.

CuO/Ag_2_WO_4_ composites were synthesized in two steps, as schematically illustrated in Fig. 1.

Initially, fluorine-doped tin oxide (FTO) glasses (thickness 2.3 mm, surface resistivity ∼7 Ω sq^−1^ and transmittance 80–82% (visible)) were spun coated with CuO seed solution, then annealed at 120 °C for 1 minute. To prepare a seed solution, 0.01 M of copper(ii) acetate monohydrate Cu(C_2_H_3_O_2_)_2_·H_2_O was dissolved in 99% methanol at 60 °C with stirring. Later, a solution of 0.03 M of potassium hydroxide (KOH) in 99% methanol was slowly added drop by drop to the copper(ii) acetate solution that was also being stirred at 60 °C. Copper precursors were prepared by mixing copper(ii) nitrate hemi(pentahydrate) (CuN_2_O_6_·2.5H_2_O) 0.05 M in 100 mL of deionized water with hexamethylenetetramine (HMT) 0.025 M. Upon preparation, a seed layer of CuO was applied through spin coating to FTO glass substrates. Substrates were then soaked in CuO solution before being baked at 80–85 °C for 5–6 hours. As a result of the growth process, a black CuO product was deposited on FTO glass substrates, indicating that CuO nanostructures were successfully formed. Additionally, using the SILAR method, silver tungstate was deposited on CuO in order to prepare composites of CuO and Ag_2_WO_4_. As part of this experiment, CuO photoelectrodes were immersed for two minutes in 0.1 M silver nitrate (AgNO_3_) solution to adsorb enough silver ions (Ag^+^), and the excess ions were then removed by submerging them in deionized (DI) water for two minutes. A second step consisted of dipping the photoelectrodes into 0.1 M sodium tungstate (Na_2_WO_4_·2H_2_O) solution for 2 minutes, and then transferring them to a DI water bath for two minutes. This dipping process is called a SILAR cycle, and the deposition of silver tungstate was performed for 5 and 10 SILAR cycles to prepare optimum Ag_2_WO_4_ nanoparticles. A final step was to improve the adhesion of Ag_2_WO_4_ by placing the photoelectrodes in an electric oven at 60 °C for 3 hours.

### 2.2. Structure, chemical composition, and morphology characterizations

We examined the morphology of fabricated photoelectrodes with a FE-SEM (Gemini 500, Zeiss) equipped with a 10 kV field emission gun. XRD was used to determine the phase identification of these samples on a Panalytical X'pert diffractometer with Cu K(α) (*λ* = 1.54 Å) 45 kV and 40 mA. We used a Linköping double Cs corrected FEI Titan3 60–300, operated at 300 kV, to perform high angle annular dark field scanning transmission electron microscopy (HAADF-STEM) imaging and EDX spectroscopy characterization. Lacey-carbon Cu TEM grids were utilized to deposit particles obtained by scratching off the sample surface. XPS with Scienta ESCA 200 and a monochromatic Al K(a) source (1486.6 eV) was employed to determine the chemical composition of CuO/Ag_2_WO_4_ composites. CasaXPS software was used to analyze the data. Following this, UV-VIS spectroscopy equipment (PerkinElmer Lambda 900 system) was used to analyze the optical properties of samples.

### 2.3. Photoelectrochemical measurements of CuO/Ag_2_WO_4_ composite

A three electrode PEC cell with a SP-200 potentiostat (Bio-Logic, Claix, France) was used to investigate the photoelectrochemical properties of the photoelectrodes. PEC cells consist of silver/silver chloride (Ag/AgCl) electrodes in 3 M KCl (reference electrode), platinum wires (counter electrodes), and fabricated electrodes (working electrodes) in 1.0 M sodium hydroxide (NaOH) as electrolyte. It was estimated that the electrode dipped in the electrolyte had an active area of 1 cm^2^. Experimental light illumination was performed using a solar simulator (LCS-100, AM 1.5G, 100 W ozone free xenon lamp). Electrochemical impedance spectroscopy (EIS) experiments were conducted at onset potentials of 0.1 V, amplitudes of 0.01 V, and sweeping frequencies of 100 kHz–1 Hz. Quantitative information about the active surface area was estimated by cyclic voltammetry.

## 3 Results and discussion

### 3.1. Structural characterization analysis of CuO/Ag_2_WO_4_ composite

**Fig. 2:**
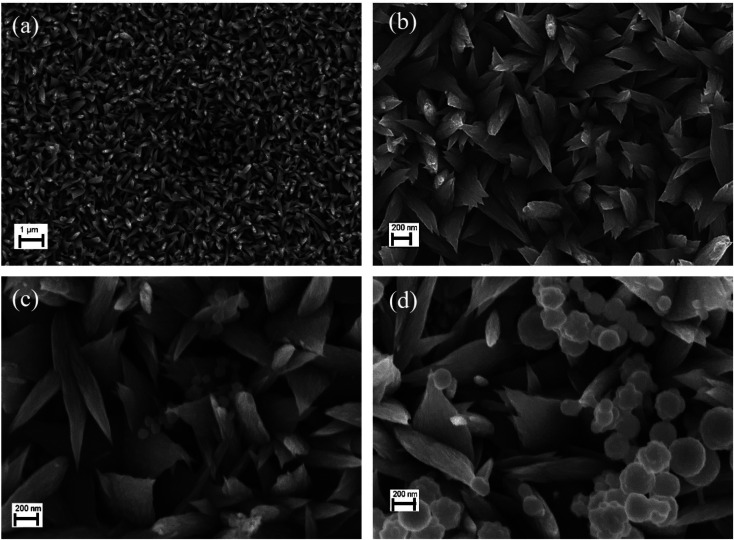
SEM images of (a), (b) pristine CuO NLs, (c) CuO/Ag_2_WO_4_ (5 SILAR cycles) and (d) CuO/Ag_2_WO_4_ (10 SILAR cycles).

In this study, FE-SEM was employed to investigate the morphology of the CuO NLs, as well as various composites of CuO/Ag_2_WO_4_ grown on FTO glass substrates. As can be seen in Fig. 2(a), the CuO photoelectrode clearly has a very dense morphology and high substrate coverage and uniformity. Using a high magnification SEM image of the CuO photoelectrode, Fig. 2(b) reveals that the nanostructures resemble nano-leaves (300–600 nm). It is confirmed from Fig. 2(c) that Ag_2_WO_4_ nanoparticles have been deposited on CuO NLs. Using a larger number of SILAR cycles, as demonstrated in Fig. 2(c) and (d), Ag_2_WO_4_ nanoparticles deposited on CuO NLs grew larger, both in size and agglomeration.

**Fig. 3:**
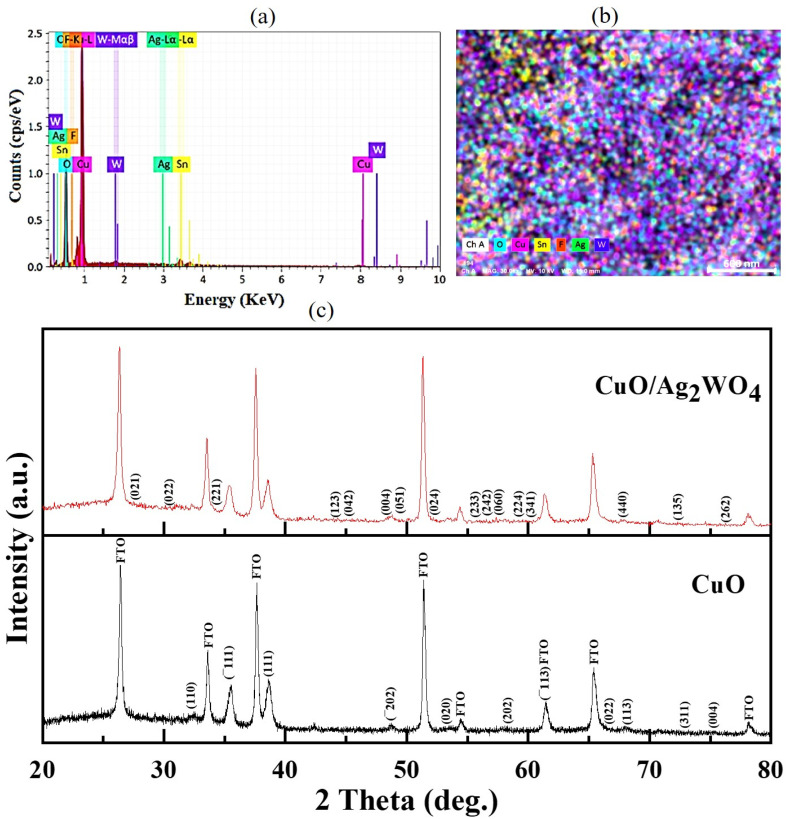
(a) EDX of the CuO/Ag_2_WO_4_ composites, (b) EDX mapping of the CuO/Ag_2_WO_4_ composites, and (c) XRD patterns of CuO NLs and CuO/Ag_2_WO_4_ composites.

EDX is shown in Fig. 3(a) and elemental mapping is shown in Fig. 3(b), confirming that Ag_2_WO_4_ nanoparticles were deposited onto CuO NLs. Chemical analysis reveals that the composites mainly consist of Cu, O, Ag, and W atoms. It can be seen from Fig. 3(a) and (b) that fluorine (F) and tin (Sn) elements arose as a result of the chemical composition of FTO glass substrate. The XRD patterns of pristine CuO NLs and various composites of CuO/Ag_2_WO_4_ deposited on FTO glass substrates are displayed in Fig. 3(c). These results confirm that all reflection peaks of pristine CuO NLs are exactly the same as those exhibited by the monoclinic phase of CuO (see JCPDS no. 96-900-8962) and the tetragonal phase of fluorine doped tin oxide (FTO). Further, after the deposition of Ag_2_WO_4_ onto the CuO NLs, some additional diffraction patterns were observed that were assigned to the hexagonal phase of Ag_2_WO_4_ (JCPDs no. 96-900-8962). In contrast to pristine CuO NLs, there is a slight shift of CuO NLs peaks in CuO/Ag_2_WO_4_ composites. This is due to morphology and size effects. Moreover, the crystallite size of synthesized samples was calculated using the Scherrer equation:^49^
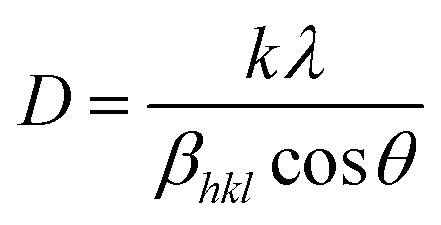
where *D* is the crystallite size, *λ* is the X-ray wavelength, *θ* is the Bragg diffraction angle, *β*_*hkl*_ is the full width at half maximum, and *k* is the Scherrer constant equal to 0.94. The average crystallite size of CuO NLs was found to decreased from 21 to 19 nm after deposition of Ag_2_WO_4_ nanoparticles.

**Fig. 4:**
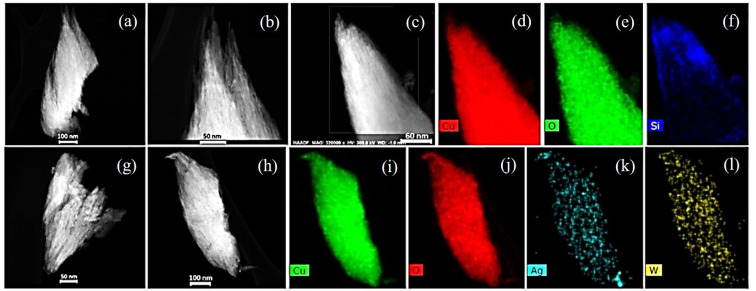
(a and b) HAADF-STEM images of CuO NLs. (c–f) HAADF-STEM image with corresponding EDX Cu, O and Si elemental maps. (g) HAADF-STEM image of CuO/Ag_2_WO_4_ particle. (h–l) HAADF-STEM image of the CuO/Ag_2_WO_4_ composites with corresponding Cu, O, Ag, and W elemental maps.

Furthermore, HAADF-STEM imaging of CuO particles (Fig. 4(a and b)) revealed that they contain crystalline fibrous filament structures. Particles were found to contain copper and oxygen as indicated by EDX spectra and maps (Fig. 4(c–e)). In some particles presence of Si was observed (Fig. 4(f)). Similar morphologies can be seen in CuO/Ag_2_WO_4_ (Fig. 4(g)). The EDX maps in Fig. 4(h–l) show an even distribution of copper, oxygen, silver, and tungsten.

**Fig. 5:**
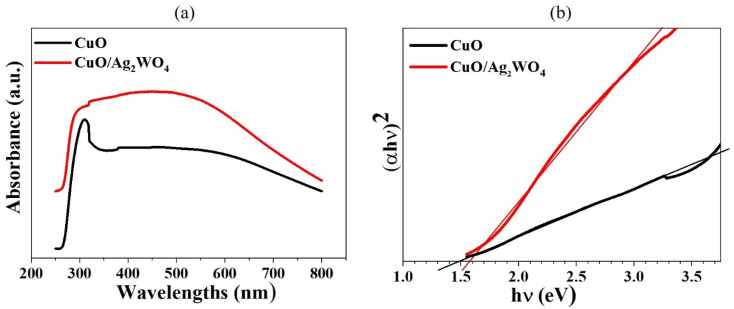
(a) The optical absorbance of the CuO and the CuO/Ag_2_WO_4_ composites, and (b) the plots of (*αhν*)^2^*versus hν*.

As shown in Fig. 5(a), UV-VIS spectroscopy was used to investigate the optical properties of pristine CuO NLs and CuO/Ag_2_WO_4_ composites. CuO/Ag_2_WO_4_ composites showed higher light absorption than pristine CuO NLs, which can help to improve HER efficiency. The optical bandgap (*E*_g_) of pure CuO NLs was measured to be 1.47 eV, while for the CuO/Ag_2_WO_4_ composite, it was approximately 1.56 eV (Fig. 5(b)). It was found that Ag_2_WO_4_ did not alter CuO's optical bandgap during deposition. Therefore, the CuO/Ag_2_W_4_ composite showed a bandgap of 1.56 eV, while pure CuO showed a bandgap of 1.47 eV. As reported in the literature, optical bandgap values range from 1.44 to 1.89 eV.^50,51^ The estimated bandgaps are optimal for the absorption of visible solar light, and they are also more conducive to splitting PEC water.

**Fig. 6:**
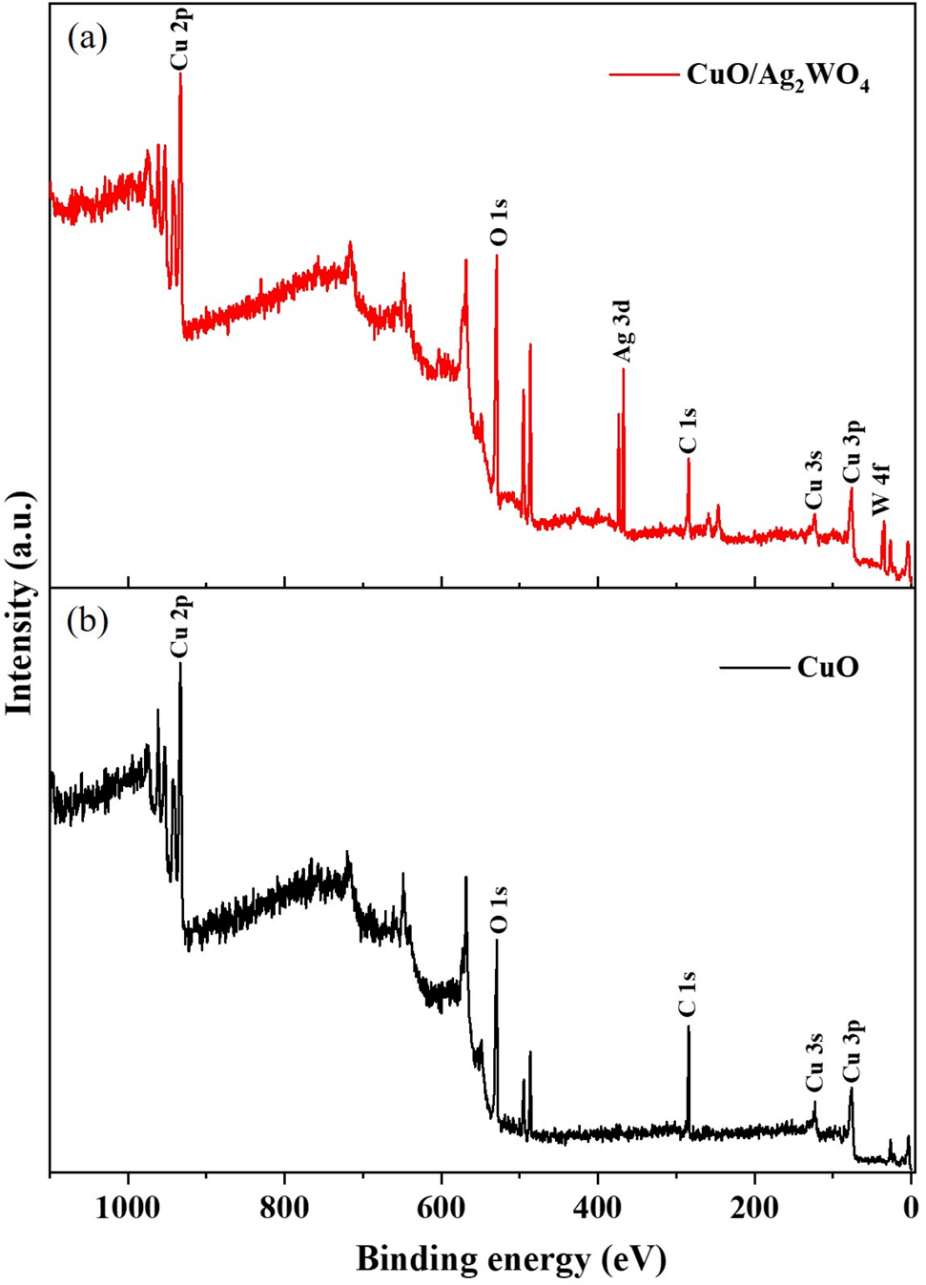
Wide scan XPS spectrum of pristine CuO NLs and CuO/Ag_2_WO_4_ composites, (a) and (b), respectively.

**Fig. 7:**
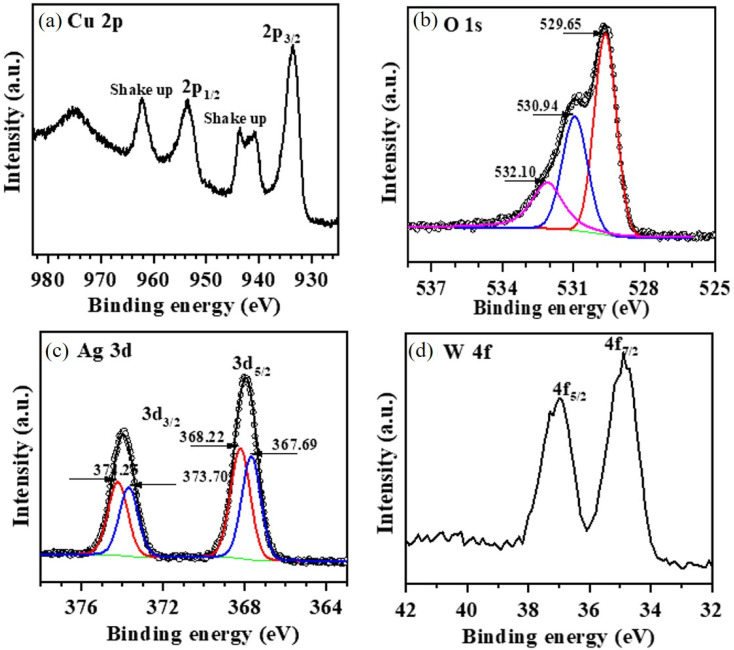
A high-resolution XPS spectrum for (a) Cu 2p spectrum, (b) O 1s spectrum, (c) Ag 3d spectrum, and (d) W 4f spectrum collected from the CuO/Ag_2_WO_4_ composites.

Furthermore, XPS analysis was carried out to understand the surface chemical features of various composites of CuO/Ag_2_WO_4_ as shown in Fig. 6. A wide scan spectrum of pristine CuO NLs and CuO/Ag_2_WO_4_ composites are shown in Fig. 6. The major peaks in CuO/Ag_2_WO_4_ composites inferred to Cu 2p, O 1s, Ag 3d, C 1s (284 eV), Cu 3s (122.5 eV), Cu 3p (76 eV) and W 4f revealing the perfect chemical composition as described by EDX. Fig. 7(a) displays high-resolution spectrum of Cu 2p. The main peaks can be assigned to Cu 2p_3/2_ (933.65 eV) and Cu 2p_1/2_ (953.55 eV) due to two natural metallic oxidation states of Cu(i) and Cu(ii). Moreover, the appearance of two satellite shake up peaks at 940.80 and 962.20 eV demonstrate the typical features of CuO.^52,53^ The deconvolution of high-resolution O 1s spectrum is shown in Fig. 7(b). The high binding region at 532.10 eV can be ascribed to the adsorbed oxygen/water species when sample is exposed to ambient condition, and the other two peaks, one for typical metal–oxygen binding energy at 529.65 eV and second is related to surface defects especially oxygen vacancies at 530.94 eV were well pronounced. Fig. 7(c) illustrates the analysis of high-resolution Ag 3d spectrum. The two peaks can be assigned to Ag 3d_5/2_ (368.05 eV) and Ag 3d_3/2_ (373.90 eV) with spin–orbit splitting difference of 6 eV, indicating the presence of Ag (0) and Ag(i) oxidation states. Furthermore, the Ag 3d spectrum can be deconvoluted into two sets of peaks based on different oxidation states. The feature (blue curve) at the lower binding energies of 367.69 and 373.70 eV can be assigned to Ag^+^ in Ag_2_WO_4_, whereas the one (red curve) at the higher binding energy at 368.22 and 374.25 eV can be assigned to metallic Ag^0^. The high-resolution W 4f spectrum is shown in Fig. 7(d). The two peaks could be associated to W 4f_7/2_ (34.90 eV) and W 4f_5/2_ (37.00 eV).^54,55^ In this sense, the XPS results confirmed the XRD and EDX results that CuO/Ag_2_WO_4_ composites had successfully been formed.

### 3.2. Photoelectrochemical analysis of CuO/Ag_2_WO_4_ composite

**Fig. 8:**
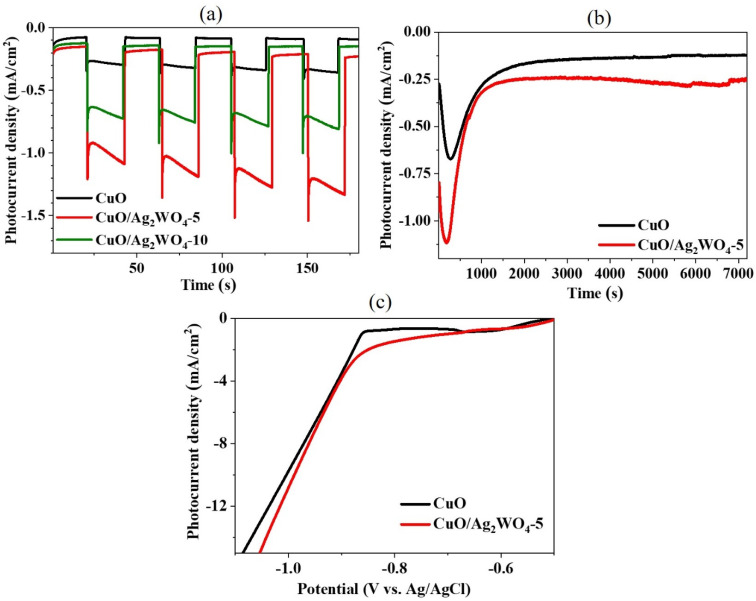
(a) The responses of CuO, CuO/Ag_2_WO_4_-5, and CuO/Ag_2_WO_4_-10 photoelectrodes to solar illumination and under ambient dark conditions, (b) the response of CuO and CuO/Ag_2_WO_4_-5 photoelectrodes for 2 hours under illumination, and (c) LSV curves under normal room light of the CuO and CuO/Ag_2_WO_4_-5 photoelectrodes.

PEC studies have been performed on both pure CuO and various CuO/Ag_2_WO_4_ composites in 1.0 M NaOH as an aqueous electrolytic solution. Fig. 8(a) displays the photocurrent density *versus* time under both solar radiation illumination and under dark conditions at a voltage of −0.55 V (*vs.* Ag/AgCl) using pristine CuO and two different CuO/Ag_2_WO_4_ composites prepared by 5 and 10 SILAR cycles labelled as CuO/Ag_2_WO_4_-5 and CuO/Ag_2_WO_4_-10, respectively. The photocurrent densities of different materials including original CuO NLs, CuO/Ag_2_WO_4_-10, and CuO/Ag_2_WO_4_-5, were observed as −0.3, −0.7, and −1.1 mA cm^−2^, respectively under solar radiation illumination and −0.09, −0.15, and −0.20 mA cm^−2^, respectively under dark conditions. It is obvious from the photocurrent density that pristine CuO is associated with low current density owing to its low density of active sites and fast charge recombination rate of electron–hole pairs during the illumination of solar light. Hence, we used the SILAR method for the improvement in the photocurrent density of CuO photocathode by depositing Ag_2_WO_4_ nanoparticles as co-catalyst. The photocurrent density of CuO/Ag_2_WO_4_-5 composite is almost fourfold higher as compared to original CuO NLs, suggesting improved photoactivity of the composite material. Furthermore, optimization was carried out in order to provide a clear picture of the composite system using 10 SILAR cycles of deposition of silver tungstate. The generation of photocurrent density was decreased from −1.1 mA cm^−2^ to −0.7 mA cm^−2^. Observations of poor performance during a higher number of 10 cycles may be attributed to the high density of Ag_2_WO_4_ onto the surface of CuO NLs. This diminishes the charge separation at the interface of the two materials, resulting in poor performance. Additionally, the 10 cycle cannot allow the high density interaction of light photons with the surface of CuO. This interaction could reach its saturation limit, which leads to the relatively low performance of this cycle. Due to their low electrical conductivity, 10 cycles of Ag_2_WO_4_ deposition decreased electron transfer and had a negative effect on PEC water splitting. PEC water splitting performance has been reported to be affected by the poor electrical conductivity of thick layer deposited films of different materials.^56^ From Fig. 8(a), the photocurrent response in cycle 10 is decreased in comparison to cycle 5. Although the difference in photocurrent between cycles 5 and 10 is not high, if we use cycles 11 and onward, then the photocurrent may be low, as observed in cycle 10. It is obvious from the difference in photocurrent between cycles 5 and 10 that the difference between cycles 6, 7, 8, and 9 could be even lower, therefore we did not study these cycles in this work. Based on the SEM images of CuO nanoleaves on the surfaces of CuO nanoleaves, it can be seen that the deposition of Ag_2_WO_4_ material is extremely low for cycles below 5. Therefore, the results indicate that such cycles as 1, 2, 3, and 4 would not produce hydrogen as effectively as cycle 5, therefore we did not report cycles such as 1, 2, 3, and 4. Photoelectrodes were subjected to a two-hour photo-corrosion test under illumination of the solar light (Fig. 8(b)). The initial photocurrent density of the pure CuO photocathode increases from −0.27 to −0.67 mA cm^−2^ within 279 seconds, but then it starts to decline and reaches −0.12 mA cm^−2^. On the other hand, the photocurrent density of the CuO/Ag_2_WO_4_ photocathode increases from −0.80 to −1.12 mA cm^−2^ during the first 194 seconds and then begins to decrease until it reaches −0.24 mA cm^−2^. It can be concluded from the stability observations that the CuO/Ag_2_WO_4_ composite generates a higher current density and is faster than pure CuO. Charge transfer between Ag_2_WO_4_ nanoparticles and CuO nanostructures is greatly facilitated by Ag_2_WO_4_ nanoparticles on CuO nanostructures. In this way, the uniform distribution of chemical elements in the hybrid system has enabled Ag_2_WO_4_ to stabilize CuO NLs in terms of charge transfer. XPS, STEM, and EDX studies have verified this conclusion. In terms of photocurrent density loss, the composite system appears to be relatively stable. In addition to stabilizing CuO, silver tungstate also improves hydrogen production performance. Furthermore, Ag_2_WO_4_ nanoparticles contribute to the increased stability of CuO/Ag_2_WO_4_-5 in part because they ensure low physical contact between CuO and the electrolyte, and they facilitate the generation of photogenerated electrons by the co-catalyst.

Using chronoamperometric response, the photocatalytic stability of CuO/Ag_2_WO_4_-5 was also evaluated under a fixed negative potential of −0.55 V (Fig. 8(b)). This graph illustrates the variation in the photocurrent density of CuO and CuO/Ag_2_WO_4_-5 hybrid materials over time. The use of the −0.55 V fixed negative potential can produce the large HER current and it avoids the electroreduction of copper ions and silver ions into their metallic atoms because their reduction potentials are even larger than the fixed negative potential of −0.55 V. Because we did not see the possibility of reducing these metallic ions, we maintained the stability of the hybrid system for an extended period of time. As compared to pristine CuO, hybrid CuO/Ag_2_WO_4_-5 material exhibits the largest photocurrent when a fixed negative potential of −0.55 V is applied, which confirms the suitability of hybrid systems for the production of hydrogen in practical applications. A linear sweep voltammetry (LSV) measurement was conducted in the presence of a normal supply of room light. Fig. 8(c) shows significant improvements in CuO/Ag_2_WO_4_ photoelectrode photocurrent density associated with the heterojunction effect associated with the deposition of Ag_2_WO_4_ nanoparticles onto CuO NLs surfaces. As a result, a large number of electrons generated by the photoelectrode are readily transferred to the counter electrode *via* the external circuit, resulting in a higher photocurrent density. Increasing the separation of charge carriers and transport efficiency, absorbing solar light at an increased rate, and increasing the absorption of visible wavelengths are the reasons for this.

In order to find the flat-band potential *V*_fb_ and free carrier concentration *N*_A_ of photoelectrodes, a Mott–Schottky (M–S) measurement was used with the following relationship:^57^
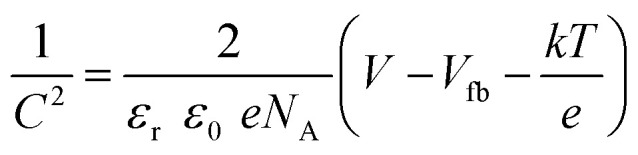
where *C* is the capacitance of the space charge layer, *ε*_T_ the relative permittivity of the semiconductor, *ε*_0_ permittivity of free space, *e* the elementary charge of an electron, *V* the applied potential, *k* Boltzmann constant, and *T* is the temperature.

**Fig. 9:**
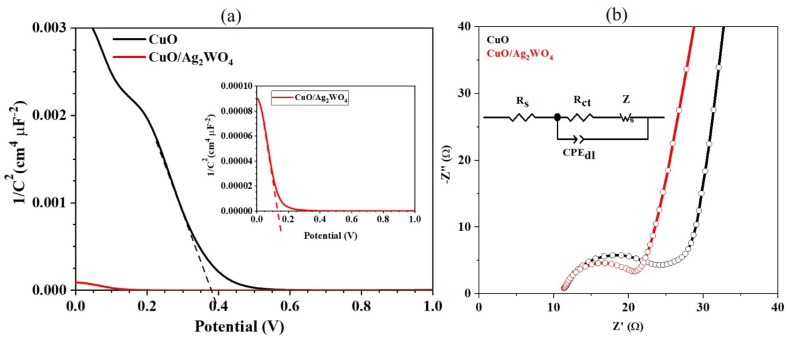
(a) The M–S plots of CuO and CuO/Ag_2_W_4_ photoelectrodes and (b) the EIS spectra acquired on CuO and CuO/Ag_2_WO_4_ photoelectrodes with equivalent circuits.

Fig. 9(a) shows the (M–S) plots for pristine CuO and the CuO/Ag_2_WO_4_-5 photoelectrodes. It is clear that both slopes of CuO and the CuO/Ag_2_WO_4_-5 photoelectrodes are negative which indicate that both materials are photocathodes (p-type semiconductors) and the space charge is populated with electrons. The flat-band potentials for pristine CuO and CuO/Ag_2_WO_4_-5 photocathodes are approximately equal to 0.38 and 0.14 V, respectively, while the free carrier concentration (the acceptor concentration in these cases) for pristine CuO and CuO/Ag_2_WO_4_-5 samples were estimated to be 6.9 × 10^20^ and 6.6 × 10^21^ cm^−3^ respectively. According to these findings, the deposition of the Ag_2_WO_4_ nanoparticles onto the CuO NLs increases the electron transfer between the photocathode and the electrolyte and lowers the redox potential at the CuO/Ag_2_WO_4_-5 surface, which results in high photoactivity towards the hydrogen evolution reaction (HER).

Charge transport phenomena were quantified using electrochemical impedance spectroscopy (EIS) to understand the reaction kinetics of HER. As shown in Fig. 9(b), the Nyquist plots for pristine CuO and pristine CuO/Ag_2_WO_4_-5 electrodes are presented. Nyquist plots with high frequencies have semicircles, while those with low frequencies have straight tails. It is generally understood that the radius of the arc indicates the magnitude of the charge-transfer resistance (*R*_ct_), while the straight tail indicates the effect of the charge diffusion procedure on the Warburg impedance (*Z*_W_) of the electrode. Using Randles–Ershler equivalent circuits to fit Nyquist plots, Fig. 9(b) illustrates such circuits.^58,59^

It is interesting to note that the CuO/Ag_2_WO_4_-5 photoelectrode in Fig. 9(b) demonstrated a significantly smaller charge transfer resistance (13.77 Ω) compared to the pristine CuO photoelectrode (18.35 Ω). A lower charge transfer resistance for the CuO/Ag_2_WO_4_ composite with 5 cycles enabled fast kinetics during the HER process. Moreover, the capacitance double layer for pure CuO and CuO/Ag_2_WO_4_-5 photoelectrode were as 0.03 and 0.01 from the EIS fitted data.

**Fig. 10:**
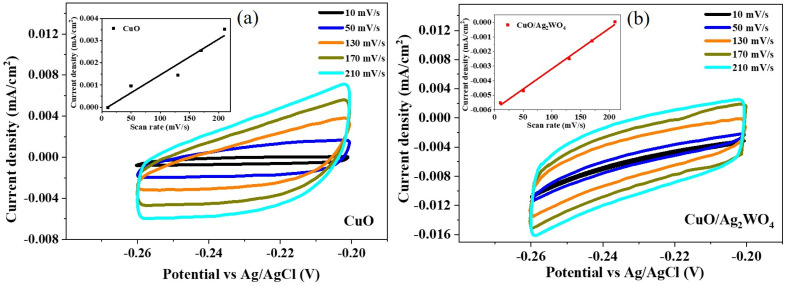
A cyclic voltammetry analysis of electrochemical active surface area and a linear fit for (a) CuO and (b) CuO/Ag_2_WO_4_ photoelectrodes.

In order to estimate the electrochemical potential surface area (ECSA), cyclic voltammetry (CV) was utilized at different scan rates as shown in (Fig. 10(a–d)). According to existing literature,^60^ CV curves at different scan rates were used to calculate the ECSA in non-faradic regions; thus, studies under non-faradic regions for the calculation of ECSA have been published. The non-faradic region of CV curves actually depicts the number of active sites present on the electrode surface that participate in electrochemical reactions. From the slope of the linear fit of the average current density *versus* the scan rate, we determined the ECSA of pure CuO (1.6 × 10^−5^ F cm^−2^) and CuO/Ag_2_WO_4_-5 (2.8 × 10^−5^ F cm^−2^). Composite catalysts have shown a high number of active sites and significantly sped up the CuO/Ag_2_WO_4_-5-based HER process. By developing an optimal interface for the acceleration of charge transport and minimizing physical contact between the CuO photocathode and electrolyte, the CuO/Ag_2_WO_4_-5 composite system achieved enhanced water splitting performance. Additionally, the architecture of the CuO NLs, tuned surface of the composite system after deposition of Ag_2_WO_4_ nanoparticles as co-catalysts, high density of active sites, and fast charge transfer rate are all factors contributing to the improved performance.

### 3.3. Photoelectrochemical HER mechanism on CuO/Ag_2_WO_4_ composite

The enhanced PEC photo-reactivity is likely caused by electron and hole separation and transfer at the photoelectrode interfaces. Electrons and holes were generated and separated much more easily in CuO/Ag_2_WO_4_-5 composites due to drastic differences in band edge potentials. A semiconductor's edge potential can be estimated using the Mulliken electronegativity theory^44^ in the valence band (VB) and in the conduction band (CB) at zero charge:

1 *E*_VB_ = *X* − *E*^*e*^ + 0.5*E*_g_

There are four parameters in this equation, whereas *E*_VB_ the VB edge potential, *X* is the absolute electronegativity of the semiconductor, *E*^*e*^ is the free electron energy on a hydrogen scale, 4.5 eV, and *E*_g_ is the band gap energy of the semiconductor. Using the following formula, the edge potential of the CB (*E*_CB_) can be calculated:

2 *E*_CB_ = *E*_VB_ − *E*_g_

**Fig. 11:**
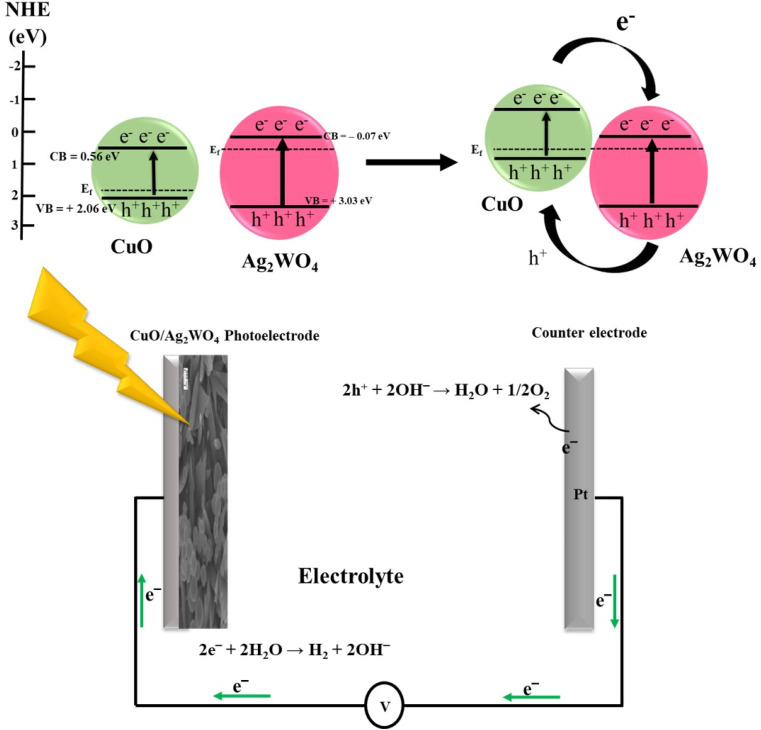
An analysis of the possible mechanisms of the CuO/Ag_2_WO_4_ photoelectrode with band edge potentials.

As a result of this equation, CuO has an *X* value of 5.81 eV and Ag_2_WO_4_ has an *X* value of 5.98 eV, and the band gaps are 1.5 and 3.1 eV, respectively.^44,61–63^ As a result, pristine CuO and Ag_2_WO_4_ have the same *E*_VBs_, which are +2.06 eV and +3.03 eV, respectively. CuO and Ag_2_WO_4_ also show similar *E*_CB_ values, both at +0.56 eV. As shown in Fig. 11, a possible mechanism underpinning PEC activity is outlined.

A CuO/Ag_2_WO_4_-5 composite is formed when CuO nanoleaves are embedded in Ag_2_WO_4_ nanoparticles, resulting in the Fermi levels aligning to achieve equilibrium.^42^ Fermi levels of CuO and Ag_2_WO_4_ coexist simultaneously because CuO has a greater Fermi potential than Ag_2_WO_4_; electrons in the Fermi levels of CuO and Ag_2_WO_4_ move toward one another until they reach equilibrium.

In the VB, light vibrates photons, which excited electrons to a higher potential. In CuO and Ag_2_WO_4_, electrons were excited to a potential of +0.56 eV and −0.07 eV, respectively. The photo-generated electrons generated by CuO produced by the CB of Ag_2_WO_4_ can move effectively to the photocathode and contribute to HER. Nanoparticles would transfer holes from their valence bands to the electrode and thus, enhance efficiency at the electrode–electrolyte interface. By limiting electron/hole recombination, this transfer enhances the efficiency of charge carrier separation while reducing electron/hole recombination. Photo-generated charge carriers are separated and transferred by this mechanism, improving the efficiency of PEC water splitting.

## 4 Conclusions

To summarize, we have synthesized an improved CuO/Ag_2_WO_4_-5 photocathode that is effective for PEC water splitting when illuminated by solar light. Samples were grown using the hydrothermal method followed by the SILAR method. FE-SEM, XRD, EDX, and XPS techniques are used to characterize the synthesized materials. CuO/Ag_2_WO_4_-5 photocathode demonstrated efficient PEC water splitting in comparison to CuO/Ag_2_WO_4_-10 photocathode and pristine CuO photocathode in alkaline media. As an alternative composite photocathode material suitable for solar-driven hydrogen production, CuO/Ag_2_WO_4_-5 composites are recommended for their low overpotential, stability, and photocurrent density. A facile, low-cost, low-emission approach to the preparation of composite materials is highly advantageous in terms of societal needs and technological advancement.

## Abbreviations

CuO: Cupric oxide
Ag_2_WO_4_: Silver tungstate
PEC: Photoelectrochemical
NRs: Nanorods
SILAR: Successive ionic layer adsorption and reaction
XRD: X-Ray diffraction
FE-SEM: Field-emission scanning electron microscope
EDX: Energy-dispersive X-ray
HAADF-STEM: High-angle annular dark-field – scanning transmission electron microscope
XPS: X-Ray photoelectron spectroscopy
LSV: Linear sweep voltammetry
M–S: Mott–Schottky

## Author contributions

Elfatih Mustafa (E. M.); writing original draft, conceptualization, prepared, characterized all samples, analyzed the data, E. A. Dawi (E. D.); writing original draft, conceptualization, investigations, Z. H. Ibupoto (Z. I.); conceptualization, writing original draft, sample analysis, A. M. M. Ibrahim (A. I.), revised draft, investigations, experimental analysis, A. Elsukova (A. E.); investigations, measurements, revised manuscript, X. Liu (X. L.); investigations, experimental analysis, sources, Aneela Tahira (A. T.); experimental analysis, sources, revised final draft, R. E. Adam (R. A.); conceptualization, investigation, sources, M. Willander (M. W.); conceptualization, writing original draft, supervision, O. Nur (O. N.); conceptualization, investigation, sources, revised final draft.

## Conflicts of interest

Authors declare no conflicts of interest.

## Supplementary Material
